# Inhibition of GALR1 in PFC Alleviates Depressive-Like Behaviors in Postpartum Depression Rat Model by Upregulating CREB-BNDF and 5-HT Levels

**DOI:** 10.3389/fpsyt.2018.00588

**Published:** 2018-11-14

**Authors:** Hui Li, Tong Wang, Cuige Shi, Yutao Yang, Xiaoxiao Li, Yan Wu, Zhi-Qing David Xu

**Affiliations:** ^1^Department of Anatomy, Capital Medical University, Beijing, China; ^2^Beijing Key Laboratory of Neural Regeneration and Repair, Beijing Laboratory of Brain Disorders (Ministry of Science and Technology), Department of Neurobiology, Beijing Institute of Brain Disorders, Capital Medical University, Beijing, China; ^3^Department of Cell Biology, National Research Institute of Family Planning, Beijing, China

**Keywords:** PPD, PFC, GALR1, CREB, BDNF, 5-HT

## Abstract

Estrogen (E2) withdrawal is a core pathology mechanism for postpartum depression (PPD). Galanin (GAL), an estrogen-inducible neuropeptide has also been reported to be associated with depression. However, it still remains unclear which GAL receptors (GALRs) are involved in PPD pathologic process. In the present study, we discovered that the expression of GALR1, rather than GALR2/3, was upregulated with a region-specific pattern in the prefrontal cortex (PFC) of E2 withdrawal induced PPD model rats. Meanwhile, c-fos was also upregulated only in PFC in the same animal model. Injection of GALR1-siRNA into the bilateral PFC ameliorated depressive-like behavior of PPD rats, suggesting that the upregulation of GALR1 in PFC is involved in PPD. Moreover, Western Blot and HPLC assays demonstrated that the downregulation of CREB-BDNF signaling and 5-HT levels in the PFC of PPD rats were reversed after GALR1-siRNA injection. These comprehensive results suggest that the knock down of GALR1 in PFC alleviates depressive-like behaviors and reverse downregulation of CREB-BDNF and 5-HT levels in PPD rat model.

**HIGHLIGHTS**

Expression level of GALR1 mRNA was significantly increased in PFC of estrogen withdraw-induced PPD rats.

Injecting GALR1-siRNA into PFC alleviated depressive-like behavior and reversed the decrease of 5-HT level and CREB/BDNF signaling in PFC of PPD rats.

## Introduction

Postpartum depression (PPD) is a severe mental disorder that affects both mother and their babies, with an estimated prevalence of 10–15% worldwide (1). The DSM-5 defines postpartum depression as the depressive episode that begins 4 weeks after delivery (2) and may last for 12 months, clinically (3). In some serious scenarios, patients may tend to commit infanticide and are more likely to abuse their babies (4). The widely accepted hypothesis of PPD is that the withdrawal of estrogen (E2) plays a cortical role in the onset of PPD (5). Clinical studies have shown that women tend to exhibit more symptoms of depression during times of large hormonal changes. Thus, the onset of PPD is thought to arise, at least in part, from the dramatic fluctuations in the levels of the gonadal hormones during the postpartum period, and patients showed strong correlation between lower estradiol level in umbilical cord blood and depressive mood during the postpartum period (4, 6). Moreover, the E2 therapy has a greater improvement in depression scores compare with placebo among patients with severe PPD. However, interfering with breastfeeding, is a major concern for E2 therapy in PPD (6, 7) and searching for a new therapeutic target is emergency for PPD treatment. Galanin (GAL) is an estrogen-inducible neuropeptide, which is widely distributed in central and peripheral nervous system, as well as endocrine system (8). It has been demonstrated that GAL system plays an important role in depression, and drugs that target galanin receptors can modulate stress-related behaviors (9–11). GAL exerts its function via its receptors: galanin receptor 1 (GALR1), galanin receptor 2 (GALR2), and galanin receptor 3 (GALR3). It's signaling via multiple transduction pathways, including inhibition of cyclic AMP/protein kinase A (GALR1, GALR3) and stimulation of phospholipase C (GALR2) (8). Our recent study revealed that the knockdown of GALR1 in the ventral periaqueductal gray reverses depressive-like behavior of chronic mild stress (CMS) rats (12). Meanwhile, it has been reported that GAL neurons in the medial preoptic area govern the parental behavior in female rats (13). Moreover, the expression of GALR1 mRNA varies across the estrous cycle in the preoptic area and is elevated in females more than males (14). However, it still remains unclear which GAL receptors (GALRs) are involved in PPD pathologic process.

In the present study, utilizing an ovarian-steroid withdrawal-induced PPD rat model, we examined the change of GALRs expression in several brain regions associated with mental disorders, including prefrontal cortex (PFC), central amygdala (CeA), and ventral hippocampus (VH). In addition, we explored whether or not there was a causal link between change of GALR1 expression and depression-like behaviors in PPD model rats and possible signaling mechanisms involved.

## Methods

### Animals and housing

Female Sprague Dawley rats (160–180 g) were used in the present study (Capital Medical University, China). To minimize the stress, the female rats were acclimatized for 1 week before ovariectomy. The rats were group housed in a room with controlled temperature (22–24°C) and light (12-h light/dark cycle). All rats had free access to food and water. The study was approved by the Animal Care Committee at Capital Medical University. It was the minimal animal number for meeting statistical analysis requirements.

### Ovariectomy

All surgeries were performed using an aseptic tip technique. Rats were anesthetized with 6% chloral hydrate (6 ml/kg) administrated i.p., and fixed at the prone position. A 1.5 cm longitudinal dorsal incision was made using an aseptic technique (15). The incision was then pulled laterally to open the muscular layer and peritoneum. The ovaries and fallopian tubes were identified and the ovaries were removed. The skin was sutured and penicillin was administered to prevent infection. The control rats were sham-operated. The rats were housed separately following surgery and allowed 1-week recovery to eliminate estrogen and progesterone.

### Procedure

#### Hormones administration

**Figure 1:**
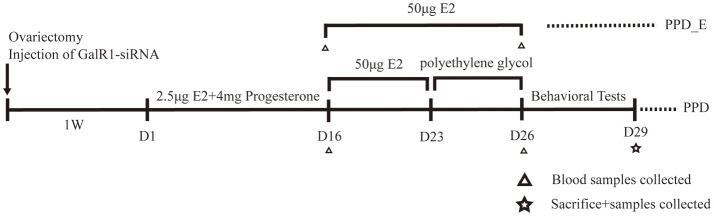
Time schedule for the experiment. Behavioral tests included forced swimming test and sucrose preference test.

The rats were divided into five groups: control group (*n* = 8), PPD group (*n* = 8), PPD + siRNA group (*n* = 8), PPD+ scramble group (*n* = 8), and PPD_E group (*n* = 8). Rats in PPD group, PPD + siRNA group, PPD+ scramble group, and PPD_E group were ovariectomized bilaterally. Rats were administrated vehicle or hormones subcutaneously at 9:00 am for 23 days from 1 week after ovariectomy. The control group was injected with 0.3 ml polyethylene for 23 days. The PPD group, PPD + siRNA group, PPD + scramble group, and PPD_E group were injected with 2.5 μg estradiol dissolved in 0.2 ml polyethylene and 4 mg progesterone (0.1 ml) for the first 16 days and 50 μg estradiol dissolved in 0.3 ml polyethylene from day17 to day23. From day 24 till to the behavioral tests were completed, rats in PPD_E group continued receiving a high dose of estradiol (50 mg), while the PPD group, PPD + siRNA group, and PPD+ scramble group were injected with 0.3 ml polyethylene during the same time. The first 23 days were considered the “pregnant” period, after which were considered the “postpartum” period (16) (Figure 1).

#### siRNA interference

The green fluorescent protein (GFP) reporting lentivirus encoding the siRNA to Galr1 and scramble (12) were injected into the bilateral PFC of rats in PPD + siRNA group or PPD + scramble group, respectively. The surgery was carried out 4 weeks before the behavioral tests. Rats were anesthetized with 6% chloral hydrate and placed on a stereotaxic apparatus (Benchmark). A hole was drilled on the skull based on the coordinate: AP+3.2 mm; ML±/−0.6 mm; DV −4.0 mm (below surface of the skull) from Bregma, according to the atlas of Paxinos and Watson (17). Three micro liters of siRNA or scramble was injected with a 10 μl microsyringe (Hamilton) for 15 min. The needle remained in place for another 15 min. The injection site was verified from 30 μm coronal sections under a fluorescence microscope. Rats were allowed one-week recovery.

### Behavior tests

#### Forced swimming test

Forced swimming test (FST) was conducted 3 days after the termination of estrogen administration in PPD group. Rats in PPD_E group were tested 2 h after the injection. The FST was carried out on rats as described in our earlier study (18), with slight modifications. Briefly, rats were individually placed in a transparent glass cylinder (64 cm height and 22 cm diameter) filled with tap water with depth of 30 cm at 25 ± 2°C. Each test session lasted for 5 min and was recorded by a camera connected to ANY- maze video tracking software (Stoelting Co., IL, USA) which automatically calculated the climbing time and immobile time. The water was changed and containers were cleaned thoroughly between rats in order to minimize any effects of other subjects. Rats were dried and returned to their cages after the test.

#### Sucrose preference test

The sucrose preference test was carried out on rats as described in our earlier study (12), All rats were trained to consume 1% sucrose solution 1 week before the test to habituate to the new solution. Animals were water-deprived 24 h before the test. The test was conducted at 11:00 a.m. 2 h following the estrogen injection in PPD_E group. Rats were placed in separated cages with no access to food. Two pre-weighed bottles were placed on each cage, one filled with tap water, the other one filled with 1% sucrose solution. The placement of two bottles (left/right) was counterbalanced and interchanged 30 min after the test started. After 1 h, bottles were re-weighted to determine the volume of the sucrose solution consumed and the sucrose preference presented by the percentage of the sucrose solution consumed.

### Radioimmunoassay for estradiol

Before the surgery and sacrifice, the rats were anesthetized with 6% chloral hydrate (6 ml/kg) administrated i.p., (at 9:00–10:00 a.m). Blood samples were collected from the retroorbital sinus using a heparan capillary tube then put into the anticoagulation tube. 20 min later, the blood was collected in Eppendorf tubes. The samples were centrifuged at 3,000 rpm (rounds per min) for 10 min. Plasma concentration of estradiol was determined by radioimmunoassay kit (Laerwen Co., LTD., Shenzhen, China) following the standard procedures.

### Microdissection of PFC, CeA, and VH sample preparation

At the end of the behavioral tests, animals were sacrificed under anesthesia. Brains were rapidly separated from the skull and sliced using a 1-mm brain matrix. PFC, CeA, and VH were dissected on ice using stereotaxic coordinates. Each brain tissue was put into an Eppendorf tube and then rapidly shock-frozen on dry ice and stored at −80°C for later RNA extraction. The positions of the cannula were verified by crystal violet staining and only the rats with an exact localization were included in the statistical analysis.

### Quantitative real-time PCR (Q-PCR)

Total RNA of brain tissues or PFC neurons was extracted using RNeasy Lipid Tissue Mini Kit (Qiagen, Germany) or RNeasy Micro Kit (Qiagen, Germany) following the manufacturer's instructions, respectively. Total RNA was dissolved in 30 μl RNase-free water provided in the kit. RNA concentrations were assessed using NanoDrop (Thermo Scientific, DE, USA) with 260 /280 nm ratios between 1.9 and 2.1. Transcriptor First Strand cDNA Synthesis Kit (Roche, IN, USA) was used to reverse-transcribe RNA (1 μg) into cDNA according to the manufacturer's instruction.

Quantitative PCR (Q-PCR) was performed following the reverse transcription. The total volume of Q-PCR reactions was 20 μl containing 8 μl distilled water, 10 μl SYBR Green Mix (Applied Biosystems, UK), 1 μl cDNA, and 1 μl primers (forward + reverse). The sequences of the primers used in this study were as followed: GAPDH-specific primers were forward: GACCACCCAGCCCAGCAAGG, reverse: TCCCCAGGCCCCTCCTGTTG. GALR1-specific primers were forward: TCGGGACAGCAACCAAAC, reverse: TGCAGATGATTGAGAACCTTGG. GALR2-specific primers were forward: GCCGCCATCGGGCTCATCTG, reverse: GTCGAGGTGCGCTCCATGCT. Amplification reaction protocol included 2 min at 60°C, 10 min at 95°C, followed by 40 cycles reaction as: 15 s of denaturing at 95°C and 1 min of annealing at 60°C. Samples were held at 10°C at the end of each amplification reaction. GAPDH was used as the internal reference for each sample.

### Western blot

Total proteins were extracted using Buffer C lysis buffer. The protein concentrations were measured by bicinchoninic acid (BCA) assay. Forty micrograms of proteins were concentrated at 80 mV and separated at 100 mV using 10% sodium dodecyl sulfate-polyacrylamide gel (SDS-PAGE). Proteins were then transferred to a polyvinylidene difluoride (PVDF) membrane (Millipore, MA, USA) for 2 h at 300 mA following the electrophoresis. Membranes were blocked with 10% milk at room temperature for 1 h and incubated with the following primary antibodies: c-fos (sc-413, mouse monoclonal, Santa Cruz CA), BDNF (ab226843, rabbit polyclonal antibody; abcome echnology), CREB and phospho-CREB (sc-377154, sc-81486, mouse monoclonal, Santa Cruz, CA) at 4°C overnight. Membranes were washed 3 times (10 min × 3) with Tris-buffered saline-Tween (TBST) and incubated with horseradish peroxidase (HRP)-conjugated secondary antibody (1:5,000, Applygen, Beijing, China) for 1 h at room temperature followed by washing 3 times (10 min × 3) with Tris-buffered saline-Tween (TBST). Proteins were developed using an enhanced chemiluminescence (ECL) reagent kit (Applygen, Beijing, China) and radiographic films (Carestream, Xiamen, China). α-tubulin (ABT170, rabbit polyclonal, 1:10,000, Millipore, Temecula, USA) was used as the internal reference.

### Determination of 5-HT and 5-HIAA levels

The levels of 5-HT and its metabolite 5-HIAA in PFC and VH was measured using high-performance liquid chromatography with anelectrochemical detector (HPLC-ECD). Model 5600A CoulArray Detector System and CoulArray for Wingdows®32 application software (ESA, USA) was used for detection and data analysis. PFC and VH tissues were dissected and stored at −80°C. Pre-weighted tissues were placed into Eppendorf tubes and homogenized in 200 μl of fluid A (0.4 M perchloric acid) for 10 s on ice. Samples were centrifuged at 12,000 rpm for 20 min at 4°C after placing on ice for 1 h, away from light. 80 μl of fluid B (mobile phase) was added into 160 μl of supernatant and vortex mixed. Samples were centrifuged at 12,000 rpm for 20 min at 4°C after placing on ice for 1 h, 200 μl of supernatant was extracted and kept at −80°C away from light. 200 μ of each sample was used for analysis. The flow rate was 1.0ml/min. The voltages of the four CoulArray channels were −150, 100, 220, and 400 mV, respectively.

### Data analyses

Data were presented as mean ± SEM (standard error of measurements).Statistical analysis was carried out using SPSS16.0. Animals with incorrect injection positions were excluded from statistical analysis. Data were analyzed with one-way ANOVA and the follow-up *post-hoc* Tukey HSD multiple comparison tests were selected to compare mean values in each group. *P* < 0.05 was considered to be statistically significant.

## Results

### E_2_ withdrawal- induced depressive-like behaviors in PPD rats

**Figure 2:**
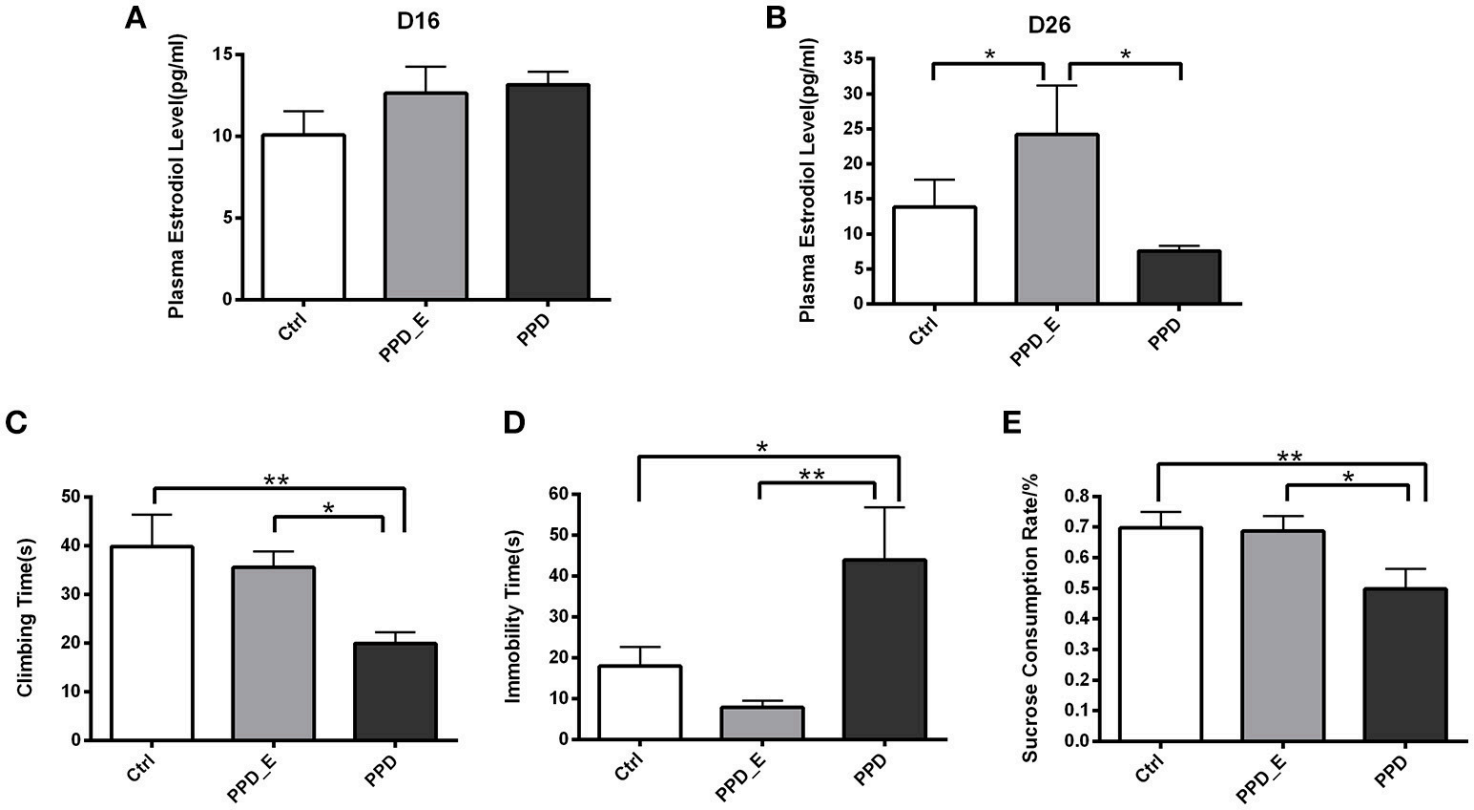
Estradiol withdrawal-induced depressive-like behaviors in PPD rats**. (A)** After the 16-day 2.5 μg E2+4 mg progesterone treatment schedule, all groups had no significant difference in plasma estradiol levels. **(B)** After the 7-day 50 μg E2 treatment schedule, PPD_E group showed a significant increase of plasma estradiol levels. **(C)** PPD group showed reduced climbing time than Ctrl and PPD_E group in forced swimming test. **(D)** PPD group showed increased immobility time than Ctrl and PPD_E group in forced swimming test. **(E)** PPD group showed a significant decrease of sucrose consumption than Ctrl and PPD_E group in sucrose preference test. Data were represented as mean ± SEM (*n* = 6–8 in each group)^*^p < 0.05 compared to PPD_E group **(A, B)**; ^*^p < 0.05, ^**^p < 0.01 compared to PPD group **(C–E)**. Data were analyzed by one-way ANOVA followed by *post-hoc* Tukey HSD multiple comparison tests.

E_2_ levels were assessed after the 16-day 2.5 μg E_2_+4 mg progesterone treatment and after the 7-day 50 μg E_2_ treatment, respectively (Figure 1). There was no significant difference in plasma E_2_ level among three groups on the 16th day (*p* > 0.05) (Figure 2A), while the E_2_ level was significantly elevated in PPD_E group after 23-day injection (*p* < 0.05) (Figure 2B).

Behavioral tests were conducted 1 week after the termination of estrogen administration in PPD group, while rats in PPD_E group kept receiving a high dose of estrogen. In forced swimming test, the climbing time was decreased in PPD group compared with the other two groups (*p* < 0.01, *p* < 0.05) (Figure 2C). However, rats in PPD group showed significantly increased immobility time (*p* < 0.05, *p* < 0.01). Sucrose consumption test showed that the PPD rats consumed less sucrose than the Ctrl rats, indicating that PPD rats performed anhedonia symptom. Meanwhile, the estrogen treatment reversed sucrose consumption to normal level in PPD_E group (*p* < 0.01, *p* < 0.05) (Figure 2E). There was no significant difference between Ctrl group and PPD_E group in forced swimming test and sucrose consumption test (Figure 2D), which is consistent with previous reports (16, 19). Maybe the dose used is not sufficient enough to influence the behavior results, though it is already higher than Ctrl.

### E_2_ withdrawal elevated GALR1 expression in PFC in PPD group

**Figure 3:**
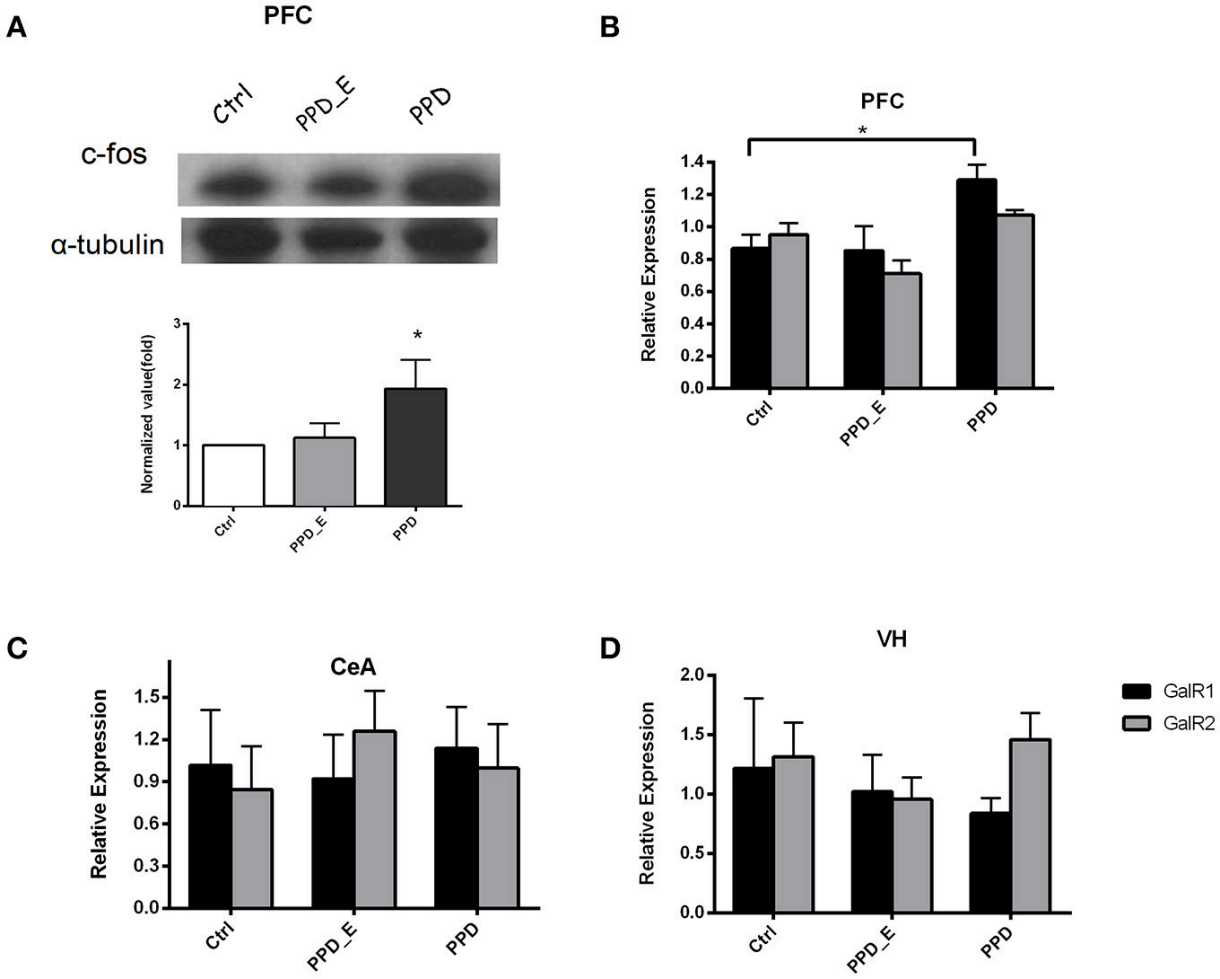
The expression of c-fos and GALR1 was significantly increased in the PFC in PPD group. **(A)** In PFC, c-fos protein level was significantly increased in PPD group compared to Ctrl and PPD_E groups. **(B)** PPD group showed an increase of GALR1 mRNA level compared to Ctrl and PPD_E group in PFC, while there was no difference in GALR2 expression between groups. **(C)** There was no difference in GALR1 or GALR2 expression between groups in CEA. **(D)** There was no difference in GALR1 or GALR2 expression between groups in VH. Data were represented as mean ± SEM (*n* = 6–8 in each group). ^*^p < 0.05 compared to Ctrl group. Data were analyzed by one-way ANOVA followed by *post-hoc* Tukey HSD multiple comparison tests.

The protein level of c-fos in PFC was significantly increased in PPD rats compared with Ctrl and PPD_E group (*p* < 0.05) (Figure 3A), but not in the VH and CeA (data not shown). The expression of GALR1 and GALR2 was analyzed in PFC, CeA, and VH brain regions. The mRNA level of GALR1 in PFC was significantly increased in PPD group compared with Ctrl and PPD_E group (*p* < 0.05) (Figure 3B), while in VH or CeA, the difference among the groups was not significant (Figures 3C,D) (*p* > 0.05). There was no difference in the expression of GALR2 in any brain regions tested (*p* > 0.05) (Figures 3B–D). It needs to be mentioned that the expression of GALR3 in rat brain is low abundance exclude hypothalamus and pituitary (20), and our data show the CT value of GALR3 in the PFC is near 37 (data not shown). Therefore, GALR3 was not analysis here.

### Injection of GALR1-siRNA into PFC reversed depressive-like behaviors in PPD rats

**Figure 4:**
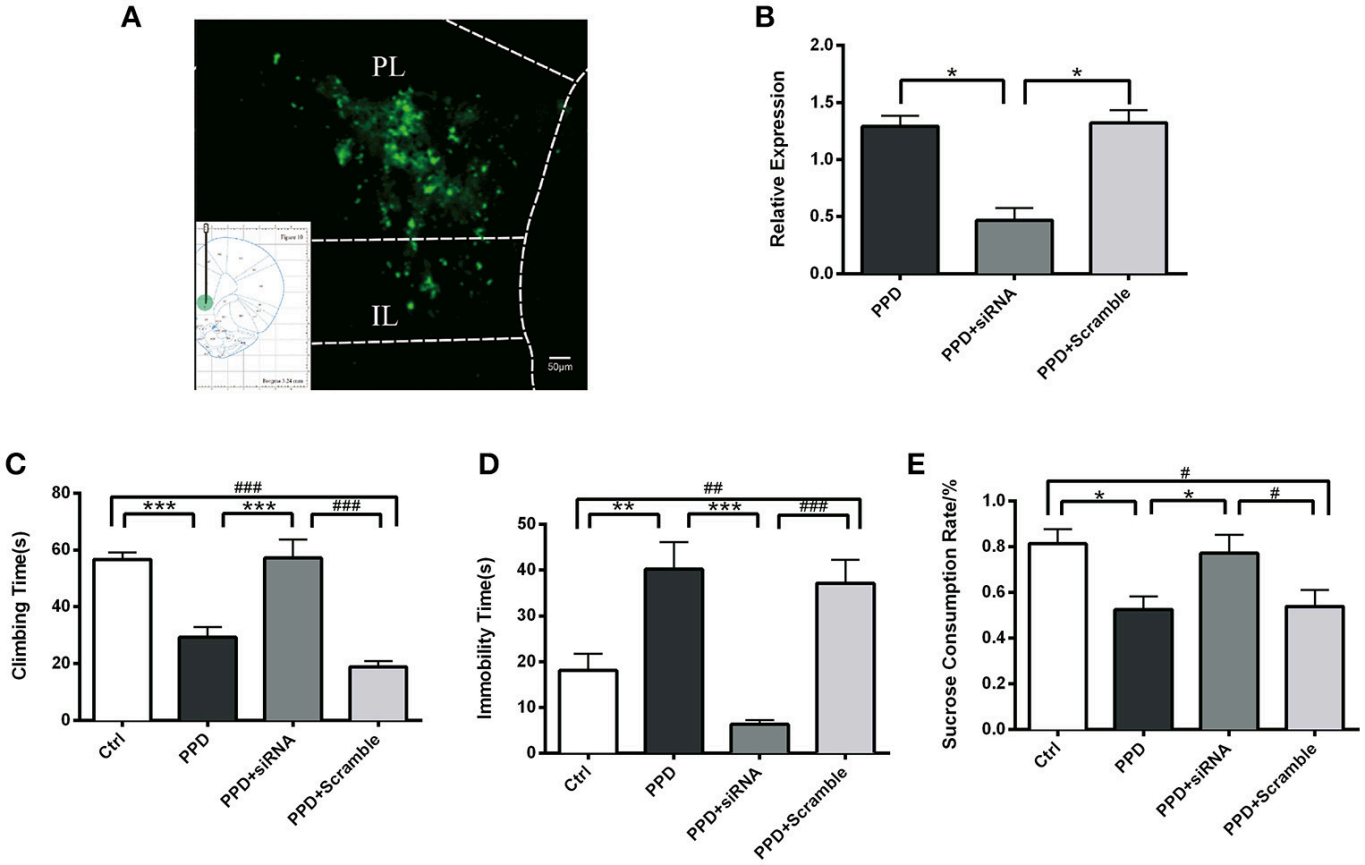
Injection of GALR1-siRNA into PFC reversed depressive-like behaviors**. (A)** A low magnification (10 ×) representative image of coronal brain sections showing the injection sites. The success of AAV vector injection into the PFC was confirmed by GFP fluorescence. **(B)** GALR1 mRNA level significantly decreased in the PFC after GALR1-siRNA treatment compared to PPD group and PPD + Scramble group. **(C)** Climbing time elevated to normal level after GALR1-siRNA treatment. **(D)** Immobility time significantly decreased in PPD + siRNA group compared to PPD groups. **(E)** Decreased sucrose consumption was reversed after GALR1-siRNA treatment. Data were represented as mean ± SEM (*n* = 6–8 in each group). ^*^p < 0.05, ^**^p < 0.01, ^***^p < 0.001 compared to PPD group; #*p* < 0.05, ##*p* < 0.01, ###*p* < 0.001 compared to PPD + Scramble group **(A–C)**. ^***^p < 0.001 compared to Ctrl group. Data were analyzed by one-way ANOVA followed by *post-hoc* Tukey HSD multiple comparison tests. Scale bar = 50 0m.

GALR1-siRNA was injected into the bilateral PFC of ovariectomized rats, following estradiol withdrawal procedure (Figure 1). The coordinates for the PFC were AP +3.2, ML ±/−0.6, H −4.0 mm from Bregma (17) and the injection sites were confirmed by GFP fluorescence (Figure 4A). The efficiency of GALR1 interference was verified by Q-PCR. The expression of GALR1 was significantly decreased in PPD + siRNA group compared with PPD and PPD + scramble groups in the PFC (*p* < 0.05), while no significant difference between PPD and PPD + scramble group was observed (*p* > 0.05) (Figure 4B).

Behavioral tests were carried out 4 weeks after GALR1-siRNA injection. In forced swimming test, PPD. and PPD + scramble rats showed less climbing time (*p* < 0.05) and more immobility time than Ctrl rats (*p* < 0.01) (Figures 4C,D), while no significant difference was seen between PPD + siRNA and Ctrl rats (*p* > 0.05) (Figures 4C,D). In sucrose preference test, PPD and PPD + scramble rats consumed less sucrose than Ctrl rats (*p* < 0.05) while GALR1-siRNA injection reversed sucrose consumption to normal level compared with Ctrl group (*P* > 0.05) (Figure 4E).

### CREB-BDNF signaling involved in the antidepressant effect of GALR1-siRNA in the PFC of PPD rats

**Figure 5:**
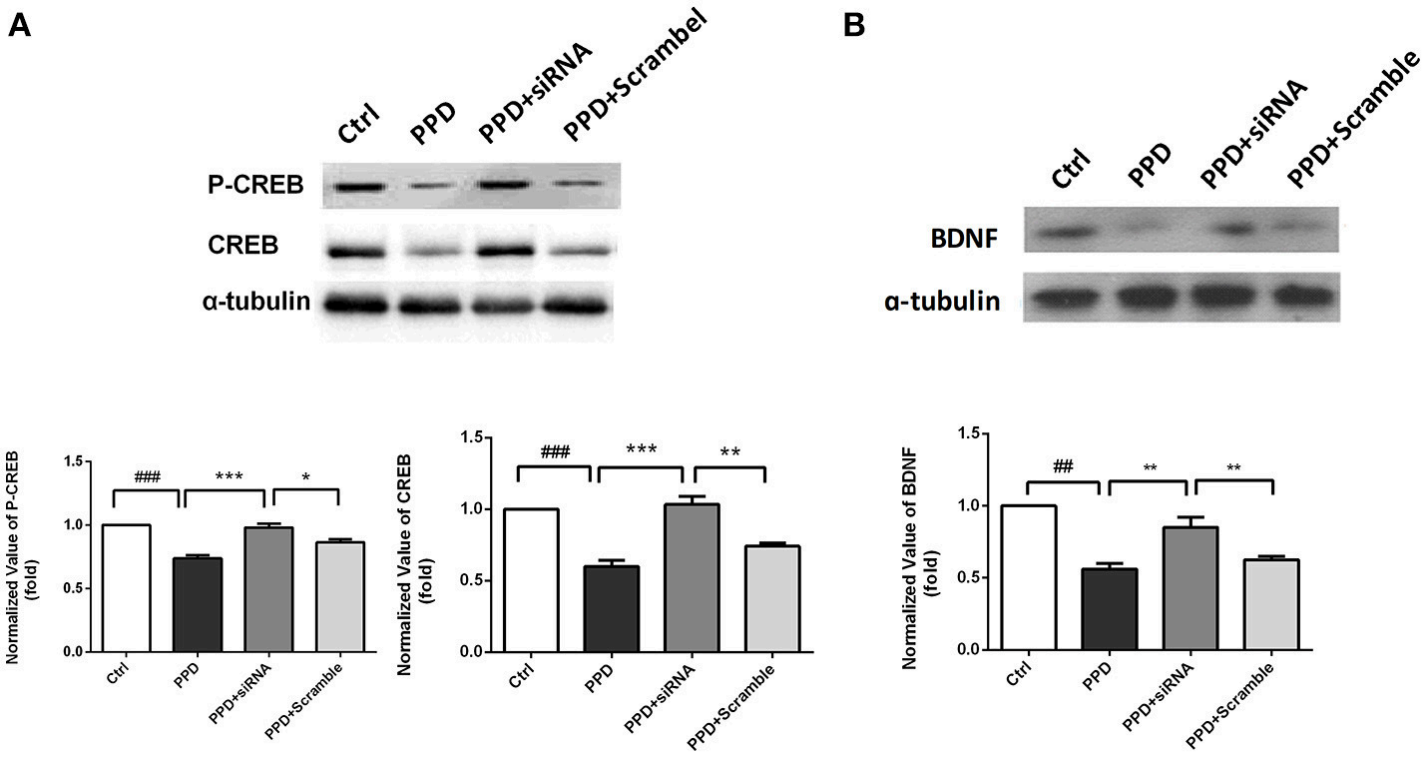
siRNA-GALR1 interference reversed the decreased protein levels of CREB, P-CREB and BDNF in the PFC. All data are expressed relative to Ctrl group.**(A)** CREB and P-CREB protein levels were significantly decreased in PPD groups, while GALR1-siRNA treatment up-regulated CREB and P-CREB levels to normal.**(B)** BDNF protein levels were significantly decreased in PPD groups, while GALR1-siRNA treatment upregulated BDNF protein levels. Values are expressed as mean ± SEM. (*n* = 3 in each group)^*^p < 0.05, ^**^p < 0.01, ^***^p < 0.001 compared to PPD + siRNA group. ##*p* < 0.01, ###*p* < 0.001 compared to Ctrl group.

CREB and phospho-CREB protein levels were assessed with WB. The expression of CREB was significantly decreased in PPD and PPD + scramble group, while GALR1-siRNA injection increased CREB expression to normal level compared to PPD group (*p* < 0.001), PPD + Scramble group, (*p* < 0.05), and Ctrl group (*p* > 0.05); The expression of P-CREB was significantly decreased in PPD and PPD + scramble group, while GALR1-siRNA injection increased phospho-CREB expression to normal level compared to PPD group (*p* < 0.001), PPD + Scramble group (*p* < 0.01) and Ctrl group (*p* > 0.05),(Figure 5A). The expression of BDNF was significantly decreased in PPD and PPD + scramble, while GALR1-siRNA injection reveres E2 withdrawal induced decreased BDNF levels compared to PPD group (*p* < 0.01), PPD + Scramble groups (*p* < 0.01), and Ctrl group (*p* > 0.05) (Figure 5B). All those data suggest that 4. CREB-BDNF signaling involved in the antidepressant effect of GALR1-siRNA in the PFC of PPD rats.

### Injection of GALR1-siRNA into PFC reversed the decreased levels of 5-HT and 5-HIAA in the PFC

**Figure 6:**
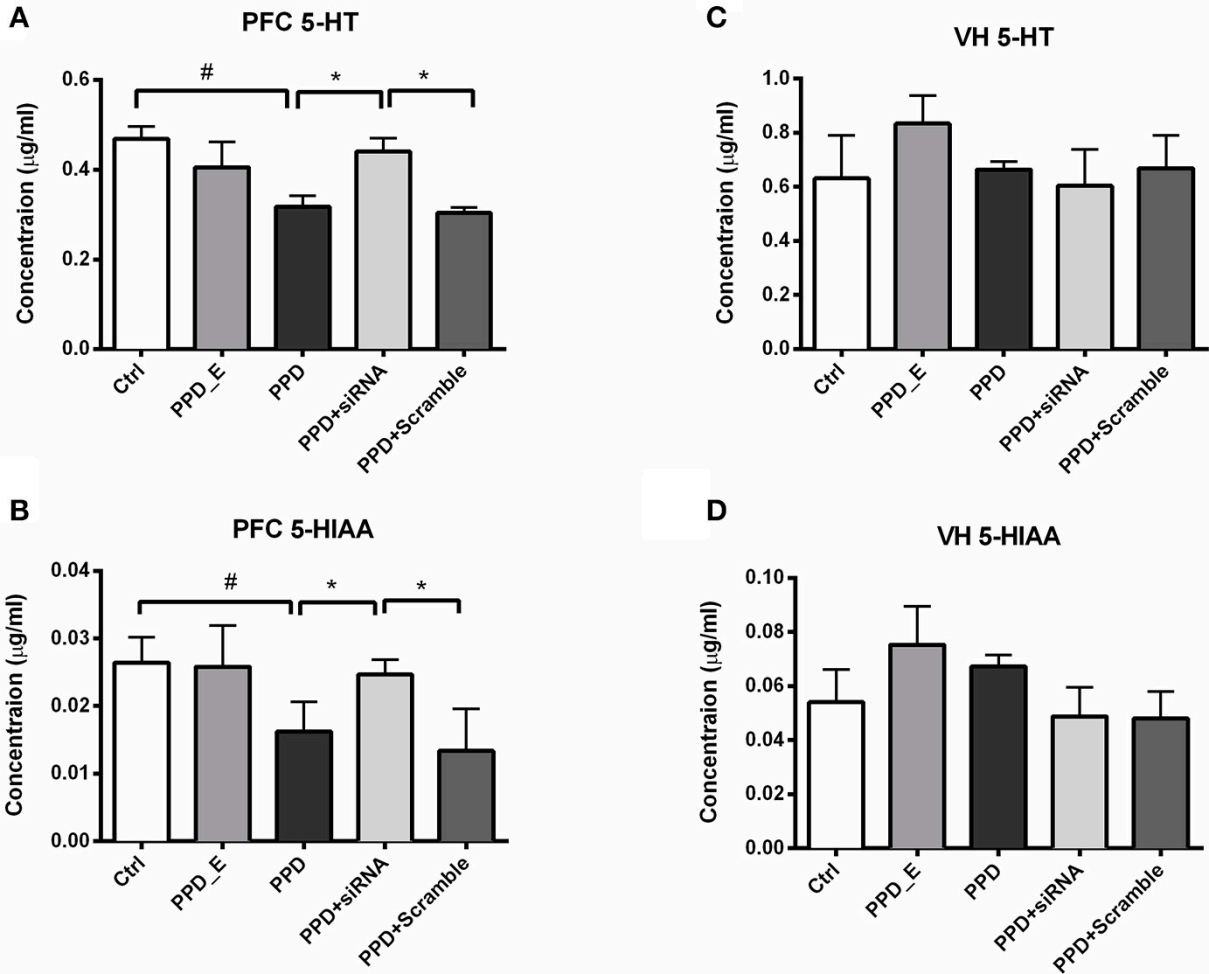
siRNA-GALR1 interference reversed down-regulation of 5-HT and 5-HIAA levels i in the PFC. **(A)** PPD + siRNA group showed significantly increased 5-HT levels in the PFC compared to Ctrl and PPD + Scramble group. **(B)** PPD + siRNA group showed significantly increased 5-HIAAlevels in the PFC compared to Ctrl and PPD + Scramble group. **(C)** There was no difference in 5-HT levels in VH between groups. **(D)** There was no difference in 5-HIAAlevels in VH between groups. Data were represented as mean ± SEM (*n* = 6–8 in each group). ^*^p < 0.05 compared to PPD + siRNA group. #*p* < 0.05 compared to Ctrl group. Data were analyzed by one-way ANOVA followed by *post-hoc* Tukey HSD multiple comparison tests. VH: ventral hippocampus.

HPLC was carried out to test the 5-HT and its metabolite 5-HIAA levels in PFC and VH. The levels of 5-HT and 5-HIAA in PFC were decreased in PPD and PPD +Scramble rats compared to Ctrl rats (Figures 6A,B). However, they were significantly increased in PPD+ siRNA rats compared to PPD and PPD +Scramble rats (*p* < 0.05). there is no significant difference between PPD+ siRNA and Ctrl group (*p* > 0.05) (Figures 6A,B). There was no significant difference of the 5-HT and 5-HIAA levels in the VH between all groups (Figures 6C,D).These results suggested that GALR1 interference may exert anti-depressant effect associated with up-regulation of 5-HT and 5-HIAA levels in the PFC.

## Discussion

In the present study, E2 withdrawal paradigm induced depressive-like behavior and upregulation of GALR1 and c-fos expression with a region specific pattern in the PFC of PPD rats. Treatment of GALR1-siRNA in PCF reversed depression-like behavior accompanied with the reversion of down-regulated CREB-BDNF and 5-HT levels.

The pathophysiology of PPD is considered to be triggered by the rapid decline in reproductive hormones following pregnancy. Besides post-partum period, lower E2 levels also associated with depression symptoms, it has been shown that CUMS-OVX rats demonstrated longer immobility time in FST test and lower sucrose preference than CUMS-intact female rats (21). However, so far most studies of depression model focus in male rats, the data about estrogen is very limited.

Consistent with other studies (16, 19), our data demonstrated that E2 withdrawal paradigm-induced depression-like behavior including decreased sugar preference, which implied this paradigm was sufficient to produce “anhedonia” in rats, suggesting that the establishment of E2 withdrawal rat model is an effective approach to investigate the mechanism of PPD. Moreover, the depressed symptoms in rodents is prevented by prolonging exposure to high levels of E2 through the “post-partum period,” suggesting that E2 plays antidepressant roles at least partially in this paradigm and further confirmed that the PPD is associated with E2 withdrawal (16). It has been shown that E2 complementary treatment plays antidepressant roles in OVX mice. However, it interferes with breastfeeding, which is a major concern in the treatment of PPD (22–24). Therefore, searching for a new therapeutic target is emergency for PPD treatment.

The monoamine-deficiency is one of the hypothesis of major depression (25). Menopausal depressive rats also showed decreased 5-HT levels in cerebral spinal fluid (26). Various studies showed that neuropeptide GAL has direct and indirect inhibitory effect on both NA and 5-HT neurons (27). It has been demonstrated that GAL system is associated with major depression in a postmortem brain study (9). Our recent study has also shown that the increased expression of GalR1 in the ventral periaqueductal gray of chronic mild stress rats is related to depression-like behavior (12). In the present study, we found that in PPD rats, GALR1 was significantly and selectively upregulated in PFC, but not in other brain regions. Meanwhile, c-fos, which is widely used as a marker for the activation of neurons in the brain (28), was also up-regulated only in the PFC in PPD rats. PFC is one of the regions involved in cortico-limbic circuits and is important in the development and treatment of depression (29). A recent proton magnetic resonance spectroscopy study showed glutamatergic dysfunction and neuronal damage occurred in dorsolateral prefrontal cortex in PPD patients (30). It has been shown that GALR1 is high expression in the mPFC (31), Taken together, all those data suggest that upregulation of GALR1 in PFC may be involved in PPD. To further demonstrate the causal link between change of GALR1 expression and depression-like behaviors in the PPD rats model, knocking down GALR1 in the bilateral-PFC with a siRNA technique was carried out and depressive-like behavior was ameliorated in PPD rats after GALR1-siRNA treatment. More interestingly, we found that higher expression of GALR1 was accompanied with lower expression of cAMP response element binding protein (CREB) and BDNF in the PFC of PPD rats. GALR1-siRNA injection reversed the decreased levels of p-CREB, CREB, and BDNF. It has been known that acitivation of GALR1, a Gi protein-coupled receptor, inhibits cyclic adenosine monophosphate synthesis and its downstream molecule CREB (31–33). Meanwhile, chronic administration of antidepressants increases the expression, phosphorylation, and function of CREB, and its downstream target gene BDNF in the limbic brain regions related to depression (29, 34, 35). All those implied that GALR1-siRNA-induced reversion of CREB-BDNF signaling might be involved in the antidepressant role of GALR1-siRNA.

Our HPLC results showed that the 5-HT and 5-HTAA levels were decreased in PFC of PPD rats. Moreover, this downregulation of 5-HT and 5-HTAA was ameliorated after GALR1-siRNA injection in the PFC. Decreased 5-HT level in the PFC associated with depression (36, 37). The concentration of 5-HT in PFC is depended on release and reuptake of 5-HT. Though it is well-known that GALR1 mRNA expresses in DR neurons (6) and very likely GALR1 protein also expresses in the terminal of DR neurons. Therefore, GALR1 might be involved in modulating release or/and reuptake of 5-HT. But local injection of GALR1-siRNA in PFC is not able to knockdown GALR1 expressed in the terminal of DR neurons as receptor protein is synthesized in the cell body. On the other hand, PFC neurons project to dorsal raphe and modulate serotonergic neurons activity as well as 5-HT releasing in target regions including PFC (38). GALR1 is highly expressed in PFC, and GALR1 mRNA level was significant increase in PFC of major depression patients (9). Thus, if the PFC-DR neurons expressing GALR1, blocking GALR1 might cause de-inhibition of PFC-DR neurons and in turn, enhance 5-HT release in PFC. However, because of lacking selective antibody against GALR1, it is still unknown whether PFC-DR neurons expressing GALR1. Meanwhile, it has been reported that astrocytes in PFC expresses 5-HT and transpoters and might be also involved in modulation of 5-HT concentration in PFC (39). It is also been reported that GALR1 expresses in astrocytes in the brain (40). Thus, local injection of GALR1-siRNA might knockdown astrocytes-expressing GALR1 and enhance release or inhibit reuptake of 5-HT. But the expression of GALR1 on astrocytes is also needed to be determined with selective antibody. In the present study, lower level of 5-HT in PPD model rats and ameliorated depression-like behavior together with reversed 5-HT level after GALR1-siRNA treatment suggesting that antidepressant effect of GALR1-siRNA might be mediated by reversing downregulation of 5-HT in PFC. Thus, GAL regulates 5-HT levels by modulating not only activity of 5-HT neurons (via GALR1/R3) in DR, the main source of 5-HT ascending system (41, 42), but also releasing or/and reuptaking of 5-HT (via GALR1) in PFC, the projection region of 5-HT ascending system.

It has been demonstrated that interaction between BDNF and 5-HT is involved in depression by influencing neuronal plasticity and depression susceptibility(43–45). Our data showed that GALR1-siRNA reversed PPD-induced downregulation of BDNF and 5-HT in PFC, suggesting that the interaction of BDNF and 5-HT might be involved in antidepressant effect of GALR1-siRNA. However, it is needed to be determined in future study.

Meanwhile, the etiology of PPD is a complex interaction of psychological, social and biological factors. It has been reported that, in addition to estrogen system, hypothalamic-pituitary-adrenal (HPA) axis disorder, gestation stress are involved in PPD (18, 46). In future study, we will continue study the effects of GAL signaling in other pathways of PPD.

## Conclusion

The present results, based on the rat PPD depression model, provide evidence for involvement of GALR1 in the PFC in depression-like behavior. Thus, a GALR1 antagonist acting in the PFC may have antidepressant actions in PPD.

## Author contributions

HL and Z-QX designed experiments. TW and CS carried out behavior and qPCR test. YY carried out WB test. XL carried out HPLC test. YW analyzed experimental results. HL and Z-QX wrote the manuscript.

### Conflict of interest statement

The authors declare that the research was conducted in the absence of any commercial or financial relationships that could be construed as a potential conflict of interest.

## References

[1] Halbreich U. Postpartum disorders: multiple interacting underlying mechanisms and risk factors. J Affect Disord. (2005) 88:1–7. 10.1016/j.jad.2005.05.002 15996747

[2] Association AP Diagnostic and Statistical Manual of Mental Disorders. 5th ed Arlington, VA: American Psychiatric Publishing (2013).

[3] Grigoriadis S VonderPorten EH Mamisashvili L Tomlinson G Dennis CL Koren G. The impact of maternal depression during pregnancy on perinatal outcomes: a systematic review and meta-analysis. J Clin Psychiatry (2013) 74:e321–41. 10.4088/JCP.12r07968 23656857

[4] Brummelte S Galea LA. Depression during pregnancy and postpartum: contribution of stress and ovarian hormones. Prog Neuropsychopharmacol Biol Psychiatry (2010) 34:766–76. 10.1016/j.pnpbp.2009.09.006 19751794

[5] Hendrick V Altshuler LL Suri R. Hormonal changes in the postpartum and implications for postpartum depression. Psychosomatics (1998) 39:93–101. 10.1016/S0033-3182(98)71355-6 9584534

[6] DelRosario GA Chang AC Lee ED. Postpartum depression: symptoms, diagnosis, and treatment approaches. Jaapa (2013) 26:50–4. 10.1097/01720610-201302000-00009 23409386

[7] Stewart DE Vigod S. Postpartum depression. N Engl J Med. (2016) 375:2177–86. 10.1056/NEJMcp1607649 27959754

[8] Lang R Gundlach AL Holmes FE Hobson SA Wynick D Hökfelt T. Physiology, signaling, and pharmacology of galanin peptides and receptors: three decades of emerging diversity. Pharmacol Rev. (2015) 67:118–75. 10.1124/pr.112.006536 25428932

[9] Barde S Rüegg J Prud'homme J Ekström TJ Palkovits M Turecki G. Alterations in the neuropeptide galanin system in major depressive disorder involve levels of transcripts, methylation, and peptide. Proc Natl Acad Sci USA. (2016) 113:E8472–81. 10.1073/pnas.1617824113 27940914 PMC5206567

[10] Kormos V Gaszner B. Role of neuropeptides in anxiety, stress, and depression: from animals to humans. Neuropeptides (2013) 47:401–19. 10.1016/j.npep.2013.10.014 24210138

[11] Picciotto MR Brabant C Einstein EB Kamens HM Neugebauer NM. Effects of galanin on monoaminergic systems and HPA axis: potential mechanisms underlying the effects of galanin on addiction- and stress-related behaviors. Brain Res. (2010) 1314:206–18. 10.1016/j.brainres.2009.08.033 19699187 PMC2819596

[12] Wang P Li H Barde S Zhang MD Sun J Wang T. Depression-like behavior in rat: involvement of galanin receptor subtype 1 in the ventral periaqueductal gray. Proc Natl Acad Sci USA. (2016) 113:E4726–35. 10.1073/pnas.1609198113 27457954 PMC4987783

[13] Wu Z Autry AE Bergan JF Watabe-Uchida M Dulac CG. Galanin neurons in the medial preoptic area govern parental behaviour. Nature (2014) 509:325–30. 10.1038/nature13307 24828191 PMC4105201

[14] Faure-Virelizier C Croix D Bouret S Prévot V Reig S Beauvillain JC, Effects of estrous cyclicity on the expression of the galanin receptor Gal-R1 in the rat preoptic area: a comparison with the male. Endocrinology (1998) 139:4127–39. 9751492 10.1210/endo.139.10.6271

[15] Kim EU Spear LP. Sex-dependent consequences of pre-pubertal gonadectomy: Social behavior, stress and ethanol responsivity. Behav Brain Res. (2016) 296:260–9. 10.1016/j.bbr.2015.09.022 26386303 PMC4659761

[16] Galea LA Wide JK Barr AM. Estradiol alleviates depressive-like symptoms in a novel animal model of post-partum depression. Behav Brain Res. (2001) 122:1–9. 10.1016/S0166-4328(01)00170-X 11287071

[17] Paxinos G Watson C The Rat Brain in Stereotactic Coordinates. San Diego, CA: Academic Press (1998).

[18] Wang T Shi C Li X Zhang P Liu B Wang H. Injection of oxytocin into paraventricular nucleus reverses depressive-like behaviors in the postpartum depression rat model. Behav Brain Res. (2018) 336:236–43. 10.1016/j.bbr.2017.09.012 28889022

[19] Stoffel EC Craft RM. Ovarian hormone withdrawal-induced “depression” in female rats. Physiol Behav. (2004) 83:505–13. 10.1016/j.physbeh.2004.08.033 15581673

[20] Smith KE Walker MW Artymyshyn R Bard J Borowsky B Tamm JA. Cloned human and rat galanin GALR3 receptors. pharmacology and activation of G-protein inwardly rectifying K+ channels. J Biol Chem. (1998) 273:23321–6. 10.1074/jbc.273.36.23321 9722565

[21] Mahmoud R Wainwright SR Chaiton JA Lieblich SE Galea LAM Ovarian hormones, but not fluoxetine, impart resilience within a chronic unpredictable stress model in middle-aged female rats. Neuropharmacology (2016) 107:278–93. 10.1016/j.neuropharm.2016.01.033 27018449

[22] Heydarpour P Salehi-Sadaghiani M Javadi-Paydar M Rahimian R Fakhfouri G Khosravi M. Estradiol reduces depressive-like behavior through inhibiting nitric oxide/cyclic GMP pathway in ovariectomized mice. Horm Behav. (2013) 63:361–9. 10.1016/j.yhbeh.2012.12.005 23262264

[23] Kandi P Hayslett RL. Nicotine and 17beta-estradiol produce an antidepressant-like effect in female ovariectomized rats. Brain Res Bull. (2011) 84:224–8. 10.1016/j.brainresbull.2010.12.012 21194557

[24] Fitelson E Kim S Baker AS Leight K. Treatment of postpartum depression: clinical, psychological and pharmacological options. Int J Womens Health (2010) 3:1–14. 10.2147/IJWH.S6938 21339932 PMC3039003

[25] Belmaker RH Agam G. Major depressive disorder. N Engl J Med. (2008) 358:55–68. 10.1056/NEJMra073096 18172175

[26] Gu S Jing L Li Y Huang JH Wang F. Stress induced hormone and neuromodulator changes in menopausal depressive rats. Front Psychiatry (2018) 9:253. 10.3389/fpsyt.2018.00253 29951006 PMC6008427

[27] Xu ZQ Zheng K Hokfelt T. Electrophysiological studies on galanin effects in brain–progress during the last six years. Neuropeptides (2005) 39:269–75. 10.1016/j.npep.2005.02.003 15944021

[28] Kovacs KJ. Measurement of immediate-early gene activation- c-fos and beyond. J Neuroendocrinol (2008) 20:665–72. 10.1111/j.1365-2826.2008.01734.x 18601687

[29] Koenigs M. Grafman J. The functional neuroanatomy of depression: distinct roles for ventromedial and dorsolateral prefrontal cortex. Behav Brain Res. (2009) 201:239–43. 10.1016/j.bbr.2009.03.004 19428640 PMC2680780

[30] Rosa CE Soares JC Figueiredo FP Cavalli RC Barbieri MA Schaufelberger MS. Glutamatergic and neural dysfunction in postpartum depression using magnetic resonance spectroscopy. Psychiatry Res Neuroimag. (2017) 265:18–25. 10.1016/j.pscychresns.2017.04.008 28494346

[31] Mitsukawa K Lu X Bartfai T. Galanin, galanin receptors, and drug targets. Exs (2010) 102:7–23. 10.1007/978-3-0346-0228-0_2 21299058

[32] O'Donnell D Ahmad S Wahlestedt C Walker P. Expression of the novel galanin receptor subtype GALR2 in the adult rat CNS: distinct distribution from GALR1. J Comp Neurol. (1999) 409:469–81. 10.1002/(SICI)1096-9861(19990705)409:3<469::AID-CNE10>3.0.CO;2-Q 10379831

[33] Hokfelt T Tatemoto K. Galanin−25 years with a multitalented neuropeptide. Cell Mol Life Sci. (2008) 65:1793–5. 10.1007/s00018-008-8152-9 18500648 PMC11131681

[34] Thome J Sakai N Shin K Steffen C Zhang YJ Impey S. cAMP response element-mediated gene transcription is upregulated by chronic antidepressant treatment. J Neurosci. (2000) 20:4030–6. 10.1523/JNEUROSCI.20-11-04030.2000 10818138 PMC6772651

[35] Suda S Segi-Nishida E Newton SS Duman RS. A postpartum model in rat: behavioral and gene expression changes induced by ovarian steroid deprivation. Biol Psychiatry (2008) 64:311–9. 10.1016/j.biopsych.2008.03.029 18471802 PMC3714803

[36] Aboul-Fotouh S. Behavioral effects of nicotinic antagonist mecamylamine in a rat model of depression: prefrontal cortex level of BDNF protein and monoaminergic neurotransmitters. Psychopharmacology (2015) 232:1095–105. 10.1007/s00213-014-3745-5 25315361

[37] Erburu M Muñoz-Cobo I Diaz-Perdigon T Mellini P Suzuki T Puerta E. SIRT2 inhibition modulate glutamate and serotonin systems in the prefrontal cortex and induces antidepressant-like action. Neuropharmacology (2017) 117:195–208. 28185898 10.1016/j.neuropharm.2017.01.033

[38] Warden MR Selimbeyoglu A Mirzabekov JJ Lo M Thompson KR Kim SY. A prefrontal cortex-brainstem neuronal projection that controls response to behavioural challenge. Nature (2012) 492:428–32. 10.1038/nature11617 23160494 PMC5929119

[39] Malynn S Campos-Torres A Moynagh P Haase J. The pro-inflammatory cytokine TNF-alpha regulates the activity and expression of the serotonin transporter (SERT) in astrocytes. Neurochem Res. (2013) 38:694–704. 10.1007/s11064-012-0967-y 23338678

[40] Hosli E Ledergerber M Kofler A Hösli L. Evidence for the existence of galanin receptors on cultured astrocytes of rat CNS: colocalization with cholinergic receptors. J Chem Neuroanat. (1997) 13:95–103. 9285354 10.1016/s0891-0618(97)00024-0

[41] Swanson CJ Blackburn TP Zhang X Zheng K Xu ZQ Hökfelt T Anxiolytic- and antidepressant-like profiles of the galanin-3 receptor (Gal3) antagonists SNAP (3788)9 and SNAP (3982)99. Proc Natl Acad Sci USA. (2005) 102:17489–94. 10.1073/pnas.0508970102 16287967 PMC1283534

[42] Xu ZQ Zhang X Pieribone VA Grillner S Hökfelt T. Galanin-5-hydroxytryptamine interactions: electrophysiological, immunohistochemical and *in situ* hybridization studies on rat dorsal raphe neurons with a note on galanin R1 and R2 receptors. Neuroscience (1998) 87:79–94. 9722143 10.1016/s0306-4522(98)00151-1

[43] Martinowich K Lu B. Interaction between BDNF and serotonin: role in mood disorders. Neuropsychopharmacology (2008) 33:73–83. 10.1038/sj.npp.1301571 17882234

[44] Gotlib IH Kasch KL Traill S Joormann J Arnow BA Johnson SL. Coherence and specificity of information-processing biases in depression and social phobia. J Abnorm Psychol. (2004) 113:386–98. 10.1037/0021-843X.113.3.386 15311984

[45] Murphy FC Sahakian BJ Rubinsztein JS Michael A Rogers RD Robbins TW. Emotional bias and inhibitory control processes in mania and depression. Psychol Med. (1999) 29:1307–21. 10.1017/S0033291799001233 10616937

[46] Dickens MJ Pawluski JL. The HPA axis during the perinatal period: implications for perinatal depression. Endocrinology (2018) 159:3737–46. 10.1210/en.2018-00677 30256957

